# Perchlorate salts confer psychrophilic characteristics in α-chymotrypsin

**DOI:** 10.1038/s41598-021-95997-2

**Published:** 2021-08-16

**Authors:** Stewart Gault, Michel W. Jaworek, Roland Winter, Charles S. Cockell

**Affiliations:** 1 UK Centre for Astrobiology, SUPA School of Physics and Astronomy, University of Edinburgh, James Clerk Maxwell Building, Peter Guthrie Tait Road, Edinburgh, EH9 3FD UK; 2 Physical Chemistry I - Biophysical Chemistry, Faculty of Chemistry and Chemical Biology, TU Dortmund University, Otto-Hahn Street 4a, 44227 Dortmund, Germany

**Keywords:** Biocatalysis, Enzymes, Biophysics

## Abstract

Studies of salt effects on enzyme activity have typically been conducted at standard temperatures and pressures, thus missing effects which only become apparent under non-standard conditions. Here we show that perchlorate salts, which are found pervasively on Mars, increase the activity of α-chymotrypsin at low temperatures. The low temperature activation is facilitated by a reduced enthalpy of activation owing to the destabilising effects of perchlorate salts. By destabilising α-chymotrypsin, the perchlorate salts also cause an increasingly negative entropy of activation, which drives the reduction of enzyme activity at higher temperatures. We have also shown that α-chymotrypsin activity appears to exhibit an altered pressure response at low temperatures while also maintaining stability at high pressures and sub-zero temperatures. As the effects of perchlorate salts on the thermodynamics of α-chymotrypsin’s activity closely resemble those of psychrophilic adaptations, it suggests that the presence of chaotropic molecules may be beneficial to life operating in low temperature environments.

## Introduction

Studies of the interactions between salts and biomolecules tend to rank their effects along the continuum of the Hofmeister series. The Hofmeister series for ions is ordered based on the ability of cations (K^+^ > Na^+^ > Li^+^ > Mg^2+^ > Ca^2+^) and anions (SO_4_^2−^ > Cl^−^ > ClO_4_^−^ > SCN^−^) at medium to high concentrations to decrease the solubility of proteins in water (‘salting-out’ effect)^1^. In general, anions appear to have a larger effect than cations. It has been proposed that salts higher up in the Hofmeister series increase the solubility of proteins in solution by weakening the strength of hydrophobic interactions ('salting-in' effect). Hence, protein stability should be disfavored with salts higher up in this series. Further, highly chaotropic anions, such as perchlorates (ClO_4_^-^), which are found pervasively on Mars^2^, have been shown to act as a protein denaturant^3^ owing to their ability to interact with the surface of biomacromolecules^4–8^. They are also able to perturb the water structure and therefore are likely to perturb hydrogen bonding, including within that of water itself^9–12^.

Chaotropic salts are normally associated with deleterious phenomena such as reduced enzyme activity and protein stability^3,13–16^, whereas kosmotropic salts normally produce the opposite effect^17,18^. However, this representation is an oversimplification as it is possible to find examples where kosmotropic salts decrease enzyme activity^19^ and examples where highly chaotropic agents increase enzyme activity^19–32^. The cases of chaotropes increasing enzyme activity raises the question as to the mechanisms of this effect.

The activation of enzymes by chaotropic molecules is frequently attributed to increased enzyme flexibility as a result of structural destabilisation^33–36^. In a folding energy landscape picture, the addition of the chaotropic salt leads to the population of a conformational substate of the native-state ensemble of conformations of the enzyme which is more suitable for substrate binding and conversion. Increased conformational flexibility is also linked to the high activity of psychrophilic enzymes at low environmental temperatures^37,38^. For psychrophilic enzymes, this increased conformational flexibility manifests thermodynamically as a lower activation enthalpy (Δ*H*^‡^) when compared to their mesophilic and thermophilic counterparts^39^. However, as psychrophilic enzymes have more conformational freedom, it means that they suffer a larger entropic penalty when forming the activated transition state, thus resulting in more negative entropy of activation values (Δ*S*^‡^)^39^.

The effect of chaotropic salts on the fluorescent quenching of proteins is also strikingly similar to what is observed with psychrophilic enzymes^19^, in which an increase in quencher penetration into hydrophobic protein regions is observed. The interpretation is that chaotropic salts disturb protein structure and that psychrophilic enzymes are more loosely packed, thus aiding quencher penetration. As psychrophilic enzymes share many similarities with non-psychrophilic enzymes exposed to chaotropic molecules, it raises the question as to whether they share similar underlying thermodynamic mechanisms. If chaotropic molecules lower enzyme activity at room temperature, but also reduce the enthalpy and entropy of activation, then we should hypothesise that this would result in increased enzyme activity at lower temperatures.

Here we use α-chymotrypsin as a model enzyme to explore the effects of perchlorate salts on enzyme activity across temperature. Bovine α-chymotrypsin is a standard serine protease which is mesophilic with regards to its temperature stability and has been shown to exhibit reduced activity and structural stability in the presence of chaotropic molecules such as perchlorate and thiocyanate salts^3,13,15,17^, whereas kosmotropic molecules such as NaSO_4_ has previously been shown to increase α-chymotrypsin’s activity and stability^17^. We show here that Mg(ClO_4_)_2_ and NaClO_4_ lower the enzyme activity of α-chymotrypsin at room temperature, but that at lower temperatures these salts become activators of enzyme activity. This low temperature activation is caused by an altered temperature dependency of enzyme activity as a result of lower Δ*H*^‡^ and Δ*S*^‡^. The results show that there is a balance between these thermodynamic parameters dictating when the low temperature activation occurs. We further show that α-chymotrypsin is pressure stable at sub-zero temperatures and that low temperatures alter the effect of pressure on the activity of α-chymotrypsin.

## Materials and methods

Lyophilised α-chymotrypsin from bovine pancreas, benzoyl-L-tyrosine ethyl ester (BTEE), D_2_O, DCl, Tris HCl and glycine were obtained from Sigma-Aldrich. Mg(ClO_4_)_2_ and NaClO_4_ were obtained from Alfa Aesar. CaCl_2_ was obtained from Fisher Scientific.

### Measuring α-chymotrypsin activity

Chymotrypsin’s activity was measured using BTEE as the substrate. Buffer consisted of 0.1 M Tris–HCl and 0.01 M CaCl_2_ adjusted to pH 7.8. Chymotrypsin concentration was maintained at 20 nM for all reactions. The concentration of the enzyme was determined using its absorbance at 280 nm with a molar extinction coefficient of 51,000 M^−1^ cm^−1^^40^. The concentrations of BTEE assayed were 10, 20, 50, 75, 100, 150 and 200 μM. Activity was measured in the absence and presence of Mg(ClO_4_)_2_ (0.25 and 0.5 M), NaClO_4_ (1 M) and glycine (1 M). Salt concentrations were selected based on results from previous work^3^ in order to ensure sufficient enzyme activity at low temperatures to differentiate the signal from noise. Glycine has been shown to increase α-chymotrypsin activity^41^ and so was included in some experiments to assess how it affected the chaotropic effect. The production of benzoyl-l-tyrosine was measured at 256 nm with a Jasco V-730 spectrophotometer during the course of the reaction. The temperature of the cuvette holder and samples were controlled and adjusted using a circulating water bath. Enzyme activity was measured from 35 to 5 °C in 5 °C increments. Measurements below 5 °C were prevented by significant condensation within the spectrophotometer which affected absorbance traces. Product formation was measured for one minute and the initial linear portion of the absorbance trace was recorded as the rate. The enzyme activity was plotted in GraphPad Prism and a least squares fit fitting method was applied according to the Michaelis–Menten equation:

1 $$v= \frac{{v}_{\mathrm{max}}\left[\mathrm{S}\right]}{{K}_{\mathrm{M}}+[\mathrm{S}]}$$

where *v*_max_ is the maximal rate, *K*_M_ is the Michaelis constant (substrate concentration at *v*_max_/2), and [S] is the substrate concentration. The high-pressure stopped-flow system, HPSF-56, from Hi-Tech Scientific was used to investigate the enzyme activity under high pressure. The enzyme concentration and substrate concentration range used, and enzymatic analysis were the same as those used for the measurements at ambient pressure. The temperature was controlled and maintained at 6 ± 0.5 °C by a thermostat. The system and function of the high-pressure stopped-flow instrument has been described in detail elsewhere^41–43^. Glycine-containing conditions were excluded from these assays due to negligible activation when BTEE is the substrate at the perchlorate concentrations assayed. The high pressure activity results have limited accuracy, however, owing to technical limitations. The spectrophotometer used has a wavelength limit of 250 nm which is close to the peak absorption wavelength of the product at 256 nm. Additionally the low operational temperatures caused the pressure cell to begin to leak above 1.5 kbar, hence limiting our ability to probe higher pressures.

### Thermodynamics of enzyme activity

The Gibbs free energy of activation (Δ*G*^‡^) was determined using transition-state theory through the following equation^44^:

2 $$\Delta {G}^{\ne }= -\mathrm{R}T \mathrm{ln}(\mathrm{h}{k}_{\mathrm{cat}}/{k}_{\mathrm{B}}T)$$

where R is the universal gas constant, h is the Planck constant, k_B_ is the Boltzmann constant, and *T* is the temperature in Kelvin. A plot of ln(*k*_cat_/*T*) vs. 1/*T* allows for the determination of the enthalpies and entropies of activation (Δ*H*^‡^, Δ*S*^‡^) from linear regressions and an expanded version of Eq. (2):

3 $${k}_{\mathrm{cat}}=({\mathrm{k}}_{\mathrm{B}}T/\mathrm{h}){\mathrm{e}}^{-\{(\Delta {H}^{\ne }/\mathrm{R}T)+(\Delta {S}^{\ne }/\mathrm{R})\}}$$

The slope of the linear regression is equivalent to − Δ*H*^‡^/R and the *y*-intercept of the regression gives Δ*S*^‡^ by (Δ*S*^‡^/R) + ln(*k*_B_/h).

### Fourier-transform infrared (FTIR) spectroscopy

For pressure-dependent Fourier-transform infrared (FTIR) spectroscopy studies, the protein was dialysed against D_2_O using Amicon Ultra centrifugation units with 10 kDa cut-off and subsequently lyophilized. The measurements were carried out in the same buffer conditions as for the HPSF measurements in the absence and in the presence of 0.25 M Mg(ClO_4_)_2_. A protein concentration of 5 wt% was used. The pD-value of both buffers was adjusted to 7.8 (pH + 0.4 = pD) by adding DCl. The temperature of the cell was regulated with an external, circulating water thermostat to − 3 °C. The equipment and setup of the high-pressure system has been described elsewhere^13^. Spectra were processed and analysed with the Grams AI 8.0 software (Thermo Fisher Scientific) as follows: after buffer subtraction and smoothing, the area of the amide I′ band was normalized to 1. The number of subbands and their positions for fitting were obtained via Fourier self-deconvolution (FSD) and 2nd derivative approaches. The amide I′ band region of α-chymotrypsin can be decomposed into eight subbands as already reported elsewhere^45^. To determine the relative changes in the population of secondary structure elements, mixed Gaussian–Lorentzian line shape functions were used to fit the peak areas in the amide I′ band region (curve fitting procedure)^46^. Assuming a two-state unfolding process of the protein, a Boltzmann function can be fitted to the temperature- and pressure-dependent sigmoidal curve progression of the intensity changes:

4 $$I=\frac{{I}_{\text{f}}-{I}_{\text{u}}}{1+{\mathrm{e}}^{-\left(\frac{1}{{T}_{\mathrm{u}}}-\frac{1}{T}\right)\cdot \frac{{\Delta H}_{\mathrm{vH}, \mathrm{u}}}{\mathrm{R}}}}+{I}_{\text{u}}$$

5 $$I=\frac{{I}_{\text{f}}-{I}_{\text{u}}}{1+{\mathrm{e}}^{-\left(p-{p}_{\mathrm{u}}\right)\cdot \frac{{\Delta V}_{\mathrm{u}}}{{\text{R}}T}}}+{I}_{\text{u}}$$

*I*_f_ and *I*_u_ are the plateau values of the IR band intensities of the folded and unfolded protein. The unfolding temperature, *T*_u_, and unfolding pressure, *p*_u_, were obtained from the inflection points of the sigmoidal curves.

### Differential scanning calorimetry

The differential scanning calorimetric (DSC) experiments were conducted using a MicroCal (Northampton, MA, USA) VP-DSC system. While the sample cell was filled with the protein solution, the reference cell contained the corresponding buffer solution with a sample volume of around 0.5 mL. A protein concentration of 1 mg mL^−1^ was used for the DSC experiments. The measurements were performed between 10 and 95 °C with a heating rate of 60 °C/h. The analysis of the DSC thermograms was carried out by the MicroCal Origin processing software. From the DSC measurements, the calorimetric enthalpy change, Δ*H*_cal_, and the thermal denaturation temperature, *T*_m_, could be obtained. Three independent calorimetric measurements revealed a calorimetric enthalpy change of 791 ± 54 kJ/mol. The value may be compromised to some extent by the high-temperature aggregation of the protein. Here, only the determination of *T*_m_ and a rough estimate of Δ*H*_cal_ is needed.

## Results

### α-Chymotrypsin activity

**Figure 1:**
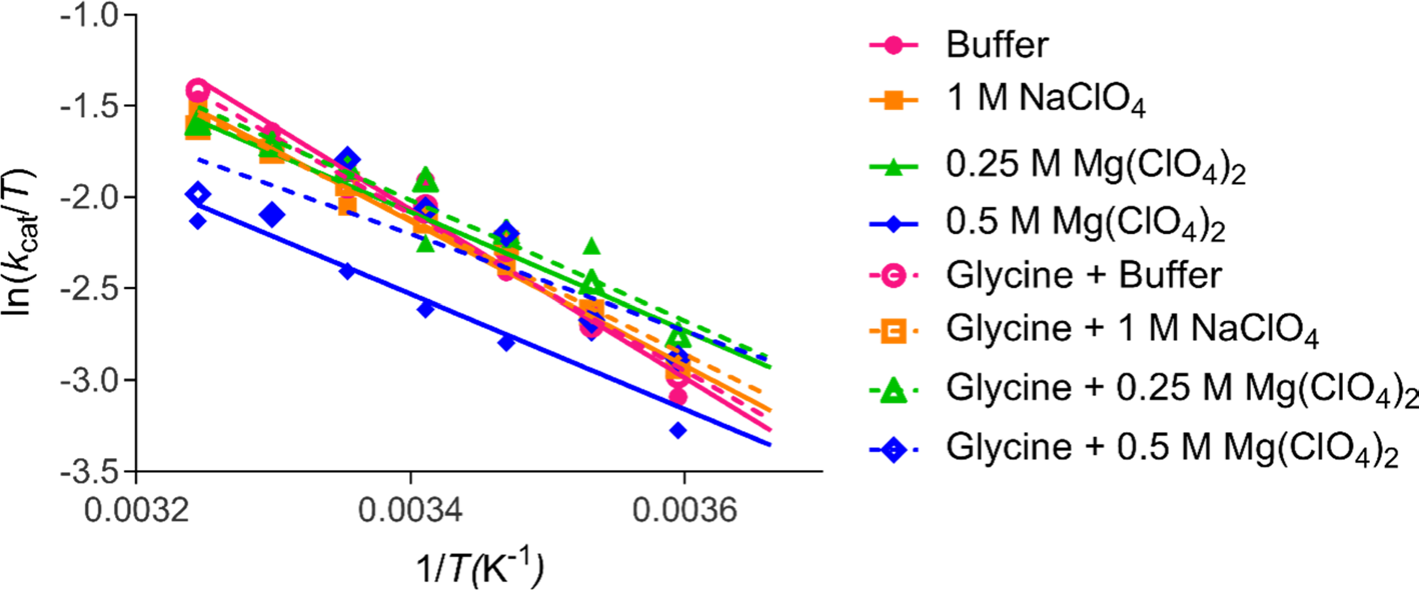
Eyring plot of α-chymotrypsin activity. The activity of α-chymotrypsin in all conditions assayed from 308 K (35 °C) to 278 K (5 °C) (left to right) with their corresponding linear regressions extrapolated from 308 to 273 K.

Figure 1 and Table 1 show that at 35 °C the activity, *k*_cat_, of α-chymotrypsin is reduced in the presence of all perchlorate-containing conditions. For example, 0.25 and 0.5 M Mg(ClO_4_)_2_ reduced the *k*_cat_ of α-chymotrypsin by ~ 10 and 35 s^−1^ respectively at 35 °C compared to the buffer only condition.

**Table 1:** α-Chymotrypsin activity values.

	*k*_cat_ (s^−1^)		*K*_M_ (μM)		*k*_cat_/*K*_M_ (M^−1^ s^−1^)	
	308 K	278 K	308 K	278 K	308 K	278 K
Buffer	71.23	12.66	72.60	24.66	9.81 × 10^5^	5.13 × 10^5^
+ Glycine	74.99	14.17	67.41	12.80	1.11 × 10^6^	1.11 × 10^6^
1 M NaClO_4_	67.47	15.02	91.49	27.49	7.37 × 10^5^	5.46 × 10^5^
+ Glycine	61.07	14.93	77.48	18.43	7.88 × 10^5^	8.10 × 10^5^
0.25 M Mg(ClO_4_)_2_	61.76	15.47	121.30	34.37	5.09 × 10^5^	4.50 × 10^5^
+ Glycine	62.55	17.73	73.40	19.35	8.52 × 10^5^	9.16 × 10^5^
0.5 M Mg(ClO_4_)_2_	36.58	10.54	54.05	4.72	6.77 × 10^5^	2.23 × 10^6^
+ Glycine	42.43	15.65	83.04	25.67	5.11 × 10^5^	6.10 × 10^5^

The turnover number (*k*_cat_), Michaelis constant (*K*_M_) and catalytic efficiency (*k*_cat_/*K*_M_) of α-chymotrypsin at 308 K (35 °C) and 278 K (5 °C) in the presence and absence of perchlorate salts and 1 M glycine. Kinetic parameters were determined from the Michaelis–Menten curves of n = 4 replicates.

As the temperature is lowered, the activities associated with different experimental conditions begin to converge, until eventually we observe increased α-chymotrypsin activity at 5 °C in the presence of perchlorate salts (except 0.5 M Mg(ClO_4_)_2_). Table 1 details the *k*_cat_ values for each condition at 5 °C. Additionally, the *K*_M_ of α-chymotrypsin was increased in the presence of perchlorate salts, with *K*_M_ gradually decreasing with increasingly lower temperatures across all conditions. The catalytic efficiency, *k*_cat_/*K*_M_, was generally lower at 35 °C in the perchlorate-containing conditions, however at 5 °C some of the perchlorate-containing conditions exhibited increased catalytic efficiency compared to the buffer only condition. The *k*_cat_, *K*_M_, and *k*_cat_/*K*_M_ of α-chymotrypsin in all tested conditions and temperatures is shown in Tables S1, S2, and S3 respectively.

The effect of the compatible solute glycine on α-chymotrypsin activity is shown in Fig. 1 and Table 1. Our results show that glycine activates α-chymotrypsin to a much lesser extent when BTEE is the substrate, compared to when *N*-succinyl-l-phenylalanine-*p*-nitroaniline is used^41^. The small activating effect of glycine may be caused by alterations to either enzyme or substrate hydration, therefore affecting the dehydration required for enzymatic catalysis. The activating effect of glycine was most evident in the 0.5 M Mg(ClO_4_)_2_ condition. In this scenario, glycine may be stabilising a subpopulation of α-chymotrypsin conformations which exhibit altered kinetic parameters compared to those present in the 0.5 M Mg(ClO_4_)_2_ condition. In general, glycine had the effect of increasing *k*_cat_, decreasing *K*_M_ and increasing *k*_cat_/*K*_M_ compared to the respective conditions without glycine.

**Figure 2:**
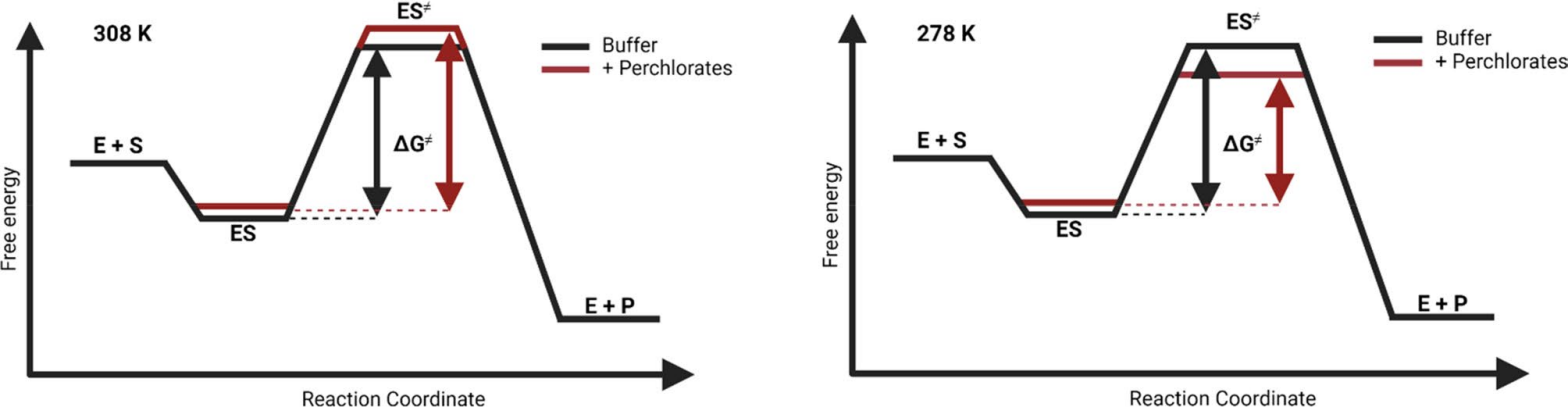
Schematic representation of the free energy changes across α-chymotrypsin’s reaction coordinate. The reaction coordinate of α-chymotrypsin at 308 and 278 K in buffer (black) and in the presence of perchlorate salts (red). Created in BioRender.com.

The thermodynamic effects of perchlorate salts and glycine on α-chymotrypsin activity are shown in Table 2. In Table 2, we observe that the increased α-chymotrypsin activity at 5 °C in the presence of perchlorate salts is derived from the lower values of Δ*G*^‡^. This is shown schematically in Fig. 2 when comparing the free energy changes of α-chymotrypsin activity at 308 and 278 K. Perchlorate salts exert this effect on α-chymotrypsin by lowering Δ*H*^‡^, which is beneficial for enzyme activity, but as Δ*S*^‡^ is also lower in these conditions, the activating effect is only seen at lower temperatures where the *T*Δ*S*^‡^ term has lesser bearing on Δ*G*^‡^. The extent to which Δ*H*^‡^ and Δ*S*^‡^ is lowered by perchlorate salts follows a standard Hofmeister series, with Mg(ClO_4_)_2_ having a greater effect than NaClO_4_. It is also concentration dependent. The general thermodynamic effect of glycine was to further decrease both Δ*H*^‡^ and Δ*S*^‡^ in most conditions, thus allowing for further enzyme activation. The Δ*G*^‡^ of all conditions tested at each temperature is shown in Table S4.

**Table 2:** α-Chymotrypsin thermodynamic parameters.

	Δ*G*^‡^ (kJ mol^−1^)		Δ*H*^‡^ (kJ mol^−1^)	Δ*S*^‡^ (J mol^−1^ K^−1^)
	308 K	278 K		
Buffer	64.63	62.07	38.21	− 84.81
+ Glycine	64.50	61.83	35.54	− 94.12
1 M NaClO_4_	64.77	61.70	32.73	− 104.01
+ Glycine	65.02	61.71	30.71	− 110.75
0.25 M Mg(ClO_4_)_2_	64.99	61.63	26.89	− 123.44
+ Glycine	64.96	61.32	27.33	− 121.22
0.5 M Mg(ClO_4_)_2_	66.34	62.52	26.13	− 129.72
+ Glycine	65.96	61.60	21.97	− 141.17

The free energy of activation (Δ*G*^‡^) at 308 K (35 °C) and 278 K (5 °C) and the enthalpy and entropy of activation (Δ*H*^‡^, Δ*S*^‡^) in the presence and absence of perchlorate salts and 1 M glycine.

**Figure 3:**
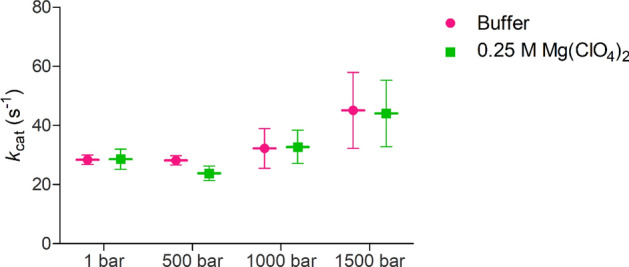
High pressure activity of α-chymotrypsin across pressure at 279 K. The high pressure enzyme activity of α-chymotrypsin expressed as *k*_cat_ at 279 K (6 °C) in buffer (pink) and in buffer containing 0.25 M Mg(ClO_4_)_2_ (orange). *N* = 4 and error bars represent the standard error of the mean.

We then attempted to analyse the relationship between temperature, pressure, and perchlorate salts on α-chymotrypsin activity in a high-pressure stopped-flow spectrophotometer (HPSF). The results are shown in Fig. 3. We found that, in the high pressure spectrophotometer (1–1500 bar), there was virtually no difference between the activity of α-chymotrypsin in absence and presence of 0.25 M Mg(ClO_4_)_2_ at 6.5 °C and that the enzyme activity remained constant across all pressures tested. The Michaelis–Menten curves for all enzyme activity measurements are shown in Figs. S1 and S2.

### Low temperature pressure stability of α-chymotrypsin

**Figure 4:**
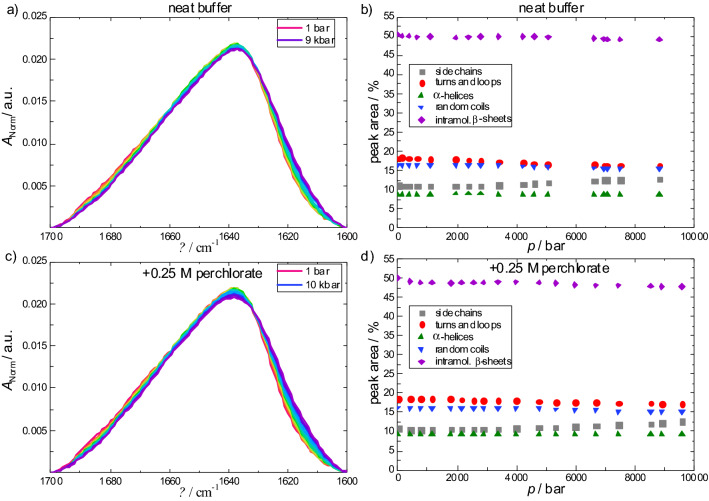
α-Chymotrypsin FTIR spectra. Pressure-dependent changes of the normalized amide I′ band of α-chymotrypsin (50 mg mL^−1^) (**a**) in neat buffer (Tris buffer + 10 mM CaCl_2_) and (**c**) + 0.25 M Mg(ClO_4_)_2_ at − 3 °C. (**b**, **d**) represent the corresponding secondary structural changes obtained from the curve fitting procedure. *N* = 3.

To explore the pressure stability of the enzyme, i.e. the stability of the secondary structure elements of the protein at − 3 °C, the amide I′ band was recorded over a pressure range from 1 bar to 10 kbar (Fig. 4). The amide I′ band of the enzyme is characterized by a broad band at 1638 cm^−1^, which shows only minor changes upon compression up to 10 kbar. Changes of the secondary structural motifs as derived from the fitting procedure of the amide I' band region (see Materials and Methods section for details) are accordingly very small (if there are any) and amount to a few percent (1–2%), only. The small shift of the amide I' band to lower wavenumbers is essentially due to an elastic compression of the protein. In the presence of 0.25 M Mg(ClO_4_)_2_ no significant changes of the amide I′ band region and the population of secondary structure elements could be observed at − 3 °C. These results demonstrate that α-chymotrypsin exhibits a remarkable stability under these high-pressure, low-temperature conditions. At higher temperatures, however, for both solution conditions, partial unfolding of α-chymotrypsin has been observed at 6–8 kbar^13^.

### *p*,* T*-stability phase diagram of α-chymotrypsin

The stability of a protein is a simultaneous function of temperature and pressure. The Gibbs free energy difference between the unfolded and native state, Δ*G* = *G*_unfolded_ − *G*_native_, with respect to some reference temperature, *T*_0_, and pressure, *p*_0_, in a second-order Taylor expansion is given by^47^:

6 $$\Delta G=\Delta {G}_{0}+\frac{\Delta {\kappa }_{T}{^{\prime}}}{2}(p-{p}_{0}{)}^{2}+\Delta \alpha {^{\prime}}(p-{p}_{0})(T-{T}_{0})-\Delta {C}_{p}\left[T\left(\mathrm{ln}\frac{T}{{T}_{0}}-1\right)+{T}_{0}\right]+\Delta V(p-{p}_{0}) -\Delta S(T-{T}_{0})$$

where ∆ denotes the change of the corresponding parameter upon unfolding, *κ'* is the isothermal compressibility factor, $\kappa_{T} ^{\prime} = \left( {\partial V/\partial p)_{T} } \right) = - V\kappa_{T}$, with ${\kappa }_{T}$ being the coefficient of isothermal compressibility, *α*′ is the thermal expansivity factor, $\alpha ^{\prime} = (\partial V/\partial T)_{p} = - (\partial S/\partial p)_{T} = V\alpha$, where *α* is the coefficient of thermal expansion of the system, ${C}_{p}$ is the heat capacity at constant pressure. The transition line, where the protein unfolds, is obtained by setting Δ*G* = 0. As reference points, we have chosen *p*_0_ = atmospheric pressure (1 bar = 10^5^ Pa) and *T*_0_ = *T*_m_, the unfolding temperature of the protein at ambient pressure. Truncation of the Taylor series at the second-order terms means that the second derivatives of the Gibbs free energy difference (Δ*C*_*p*_, Δ*κ'*, Δ*α*′) do not change significantly with temperature and pressure. As can be seen from [Eq. (6)], for calculating ∆*G*, experimental data for all input parameters are required. For selected monomeric proteins, such as the protein staphylococcal nuclease (SNase), all thermodynamic input parameters have been determined experimentally^47^, enabling us to successfully calculate ∆*G*(*p,T*) and the corresponding *p*, *T*-stability diagram of the protein. For α-chymotrypsin, available experimental data for *T*_m_ and Δ*H* have been used. For the remaining parameters, reasonable values have been assumed or were derived by the fitting procedure to the experimental data points: α-chymotrypsin in buffer solution: *T*_m_ = 329.1 K (FTIR data: 330.7 K, DSC data: 327.5 K), Δ*H* = 800 kJ mol^−1^ (DSC data: 791 ± 54 kJ mol^−1^), Δ*V* = − 35 mL mol^−1^, Δ*C*_*p*_ = 3.5 kJ mol^−1^ K^−1^, Δ*α* = 7.5 × 10^−3^ K^−1^, Δ*κ* = 3.0 10^–7^ bar^−1^; α-chymotrypsin in 0.25 M Mg(ClO_4_)_2_: *T*_m_ = 318.6 K, Δ*H* = 750 kJ mol^−1^, the other parameters have been kept constant).

**Figure 5:**
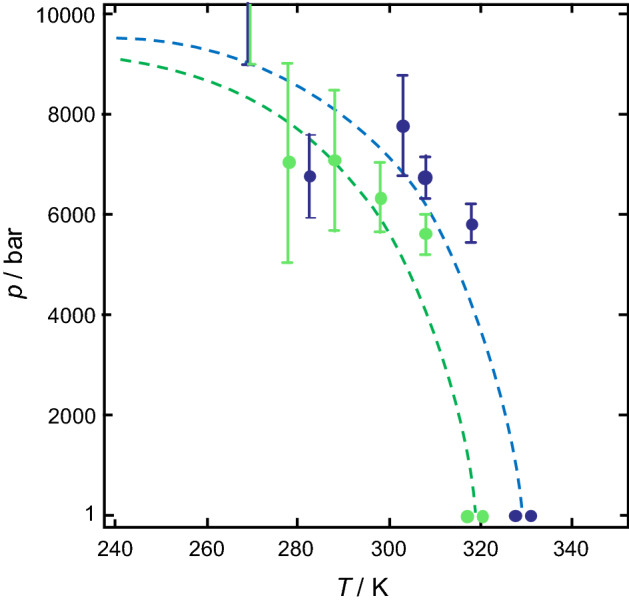
Pressure–temperature stability diagram of α-chymotrypsin. The pressure–temperature stability diagram of α-chymotrypsin in pure buffer (blue data points and dashed line) and in 0.25 M Mg(ClO_4_)_2_ (green data points and dashed line). Calculated data (dashed lines) were derived from the contours of the Gibbs free energy of unfolding at ∆*G* = 0 [Eq. (6)]. Data points indicate measured values at different temperatures and pressure obtained from FTIR spectroscopy (at ambient and high pressure) or DSC (at 1 bar). Error bars show the maximum error and are derived from at least two independent measurements, *N* ≥ 2.

Figure 5 shows the *p*,*T*-phase diagram for α-chymotrypsin in pure buffer and in 0.25 M Mg(ClO_4_)_2_, respectively (the two *T*_m_-values at ambient pressure correspond to data from FTIR spectroscopy and DSC measurements, respectively). The lines show the fits to the experimental data using the parameters given above. Note that, owing to the many parameters involved, slightly different parameter sets will lead to similar fits. Further, as the unfolding process of α-chymotrypsin may not obey a simple two-state unfolding scenario at this pH value and solution conditions, van't Hoff-derived enthalpy changes from spectroscopic data may differ significantly from the calorimetric ones. Further improving the fits to obtain better fits by assuming temperature and pressure dependent Δ*C*_*p*_, Δ*α* and Δ*κ* values was omitted here. Hence the calculated stability curves may be regarded a rather approximate.

## Discussion

Enzymes are adapted to operate across a range of chemical and physical parameters encountered in their environment. The effects of such parameters on enzyme activity and stability are normally studied in isolation. This, however, risks obscuring how environmental parameters collectively come together to affect enzymes and shape their evolution. Here we have shown that perchlorate salts alter the thermodynamic properties of α-chymotrypsin activity, and as a result, alters the temperature dependency of its activity. In essence, perchlorate salts impart psychrophilic characteristics unto bovine α-chymotrypsin.

We observed that α-chymotrypsin activity was increased at low temperatures in the presence of perchlorate salts and showed that this was caused by the salt lowering the activation enthalpy. As perchlorate salts destabilise enzymes, their disruptive effect reduces the number of weak stabilising interactions (e.g., H-bonds), thus increasing the conformational flexibility of the enzyme. The reduced number of stabilising interactions means that fewer bonds need to be broken in order to form the transition state complex, resulting in a lower activation enthalpy, which is beneficial for enzymatic catalysis. Previous studies on the activation of enzymes by chaotropic molecules have also suggested that such activation is caused by increased conformational flexibility^33–36^, as evidenced by increased fluorescent quencher penetration^19^ and an increased susceptibility to tryptic digest^23^. Furthermore, recent elastic incoherent neutron scattering experiments have shown that chaotropic agents, such as urea, increase the fast sub-nanosecond dynamics (mean-squared displacements) of protein atoms within the native fold^48^. Certainly, if the perchlorate concentration is too high, partial, or full unfolding of the enzyme will take place, which will cause the enzyme activity to be lost.

Other mechanisms may contribute to the increase in enzyme activity. Owing to the chaotropic nature of the perchlorate anion at the high concentrations we tested, the hydrogen-bond network structure of the solvent H_2_O is perturbed^9–12^, which might change the hydration properties of the reactants (and hence their activities), and, for example, help facilitate dehydration of the substrate and active site in the course of the reaction. Additionally, the extent to which α-chymotrypsin is preferentially hydrated may differ across temperatures when in the presence of solutes such as perchlorate salts and glycine. As such, preferential hydration of α-chymotrypsin at low temperatures may contribute to the increased enzymatic activity observed. However, as we lack direct data pertaining to the extent to which α-chymotrypsin is preferentially hydrated across the various conditions assayed, we cannot directly ascribe the observations made here to preferential hydration. As a thermodynamic analysis is possible with the results obtained, we are obliged to interpret our results through the thermodynamic parameters and reference how such thermodynamic changes have been interpreted in previous studies of chaotropic salts and psychrophilic enzymes.

The increased conformational flexibility of α-chymotrypsin induced by perchlorate salts does, however, come with a price, namely an increasingly negative entropy of activation. That is to say that in the presence of perchlorate salts, there is a greater loss of entropy in forming the highly ordered transition state complex from the enzyme–substrate complex. In fact, in a volumetric analysis of α-chymotrypsin-catalysed peptide hydrolysis reactions, we could show that the transition state (ES^≠^) is rather compact and has a smaller partial molar volume compared to the ES complex^41^. As the magnitude of the entropic effect on enzyme catalysis is temperature dependent, -*T*Δ*S*^‡^, we see different effects of perchlorate salts on α-chymotrypsin activity at different temperatures. At higher temperatures when the entropic contribution is larger, we observe that perchlorate salts lower α-chymotrypsin activity. However, when the temperature is lower, and the entropic contribution is much smaller, the benefit of the reduced activation enthalpy can be realised, resulting in increased enzyme activity.

The delicate balance between the enthalpic and entropic contributions is reflected in the Gibbs free energy of activation values at each temperature. At higher temperatures it was the buffer conditions which exhibited the lowest Δ*G*^‡^ values, whereas at lower temperatures it was the perchlorate salt-containing conditions which had the lowest Δ*G*^‡^. It is also an important reminder that, owing to the exponential dependence of *k*_cat_ on Δ*G*^‡^ [Eq. (2)], it only requires small changes to the Δ*G*^‡^ of an enzyme in order to facilitate large changes in activity.

The effect that we observed of perchlorate salts on α-chymotrypsin’s thermodynamics, kinetics, and stability, are analogous to those seen when comparing the behaviour of psychrophilic enzymes to mesophilic enzymes. Namely, psychrophilic enzymes exhibit lower values of Δ*H*^‡^ and Δ*S*^‡^, increased low temperature activity, and reduced thermal stability compared to mesophilic enzymes^39^.

Our results have some implications for natural environments. As the effects of chaotropic perchlorate salts mimic psychrophilic adaptations, the data show how, in theory, adaptation to the presence of perchlorate salts could also enable life to operate, or transition more easily into, low temperature conditions. We note the intriguing implications for Mars, a planet with pervasive perchlorate salts in its crust^2^, which in the near surface and deep-subsurface, experiences low temperatures. Our data show that perchlorate salts and low temperatures complement each other in terms of the theoretically required biochemical adaptations, rather than representing two separate deleterious extremes, with implications for the habitability of high perchlorate-low temperature Martian environments. The concept that chaotropic molecules aid in low temperature survival has been proposed previously^49^, here we have demonstrated a thermodynamic effect which may be the underlying reason for why chaotropic molecules support low temperature growth.

The phase diagram produced from the combination of DSC and FTIR shows that α-chymotrypsin is stable at all temperatures and pressures assayed, and that it remains stable at sub-zero temperatures. We had hoped to examine the thermodynamic phase diagram of α-chymotrypsin across temperature, pressure and Δ*G* conditions. However, the thermogram from α-chymotrypsin in the presence of perchlorate salts exhibited multiple peaks, so while the enzyme clearly unfolds, the irreversibility of the process prevented thermodynamic interpretation. This illustrates the technical challenges encountered when employing standard biophysical and biochemical techniques to biochemistry under extreme conditions. Surmounting such limitations will eventually allow for a detailed molecular and physical understanding of the biophysical limits to life.

In conclusion, it has been demonstrated for the first time that an enzyme can be activated at low temperatures by a chaotropic salt which has historically only been thought of as purely deleterious with regards to enzyme activity. We demonstrate that this effect is achieved through a reduction of the activation enthalpy, and that the temperature at which this activating effect becomes apparent is largely determined by the magnitude of the entropy of activation.

## Supplementary Information

Supplementary Information 1.

Supplementary Information 2.

## Data Availability

The source data for the figures presented in this study have been included as a supplementary file.
