# Supplementary material for: Perchlorate salts confer psychrophilic characteristics in α-chymotrypsin
Source: Sci Rep. 2021 Aug 16;11:16523. doi: 10.1038/s41598-021-95997-2 (PMC8367967; doi:10.1038/s41598-021-95997-2)
Supplement: Supplementary file 2 — Supplementary Information 2. [file 41598_2021_95997_MOESM2_ESM.docx]

**Supplementary information**


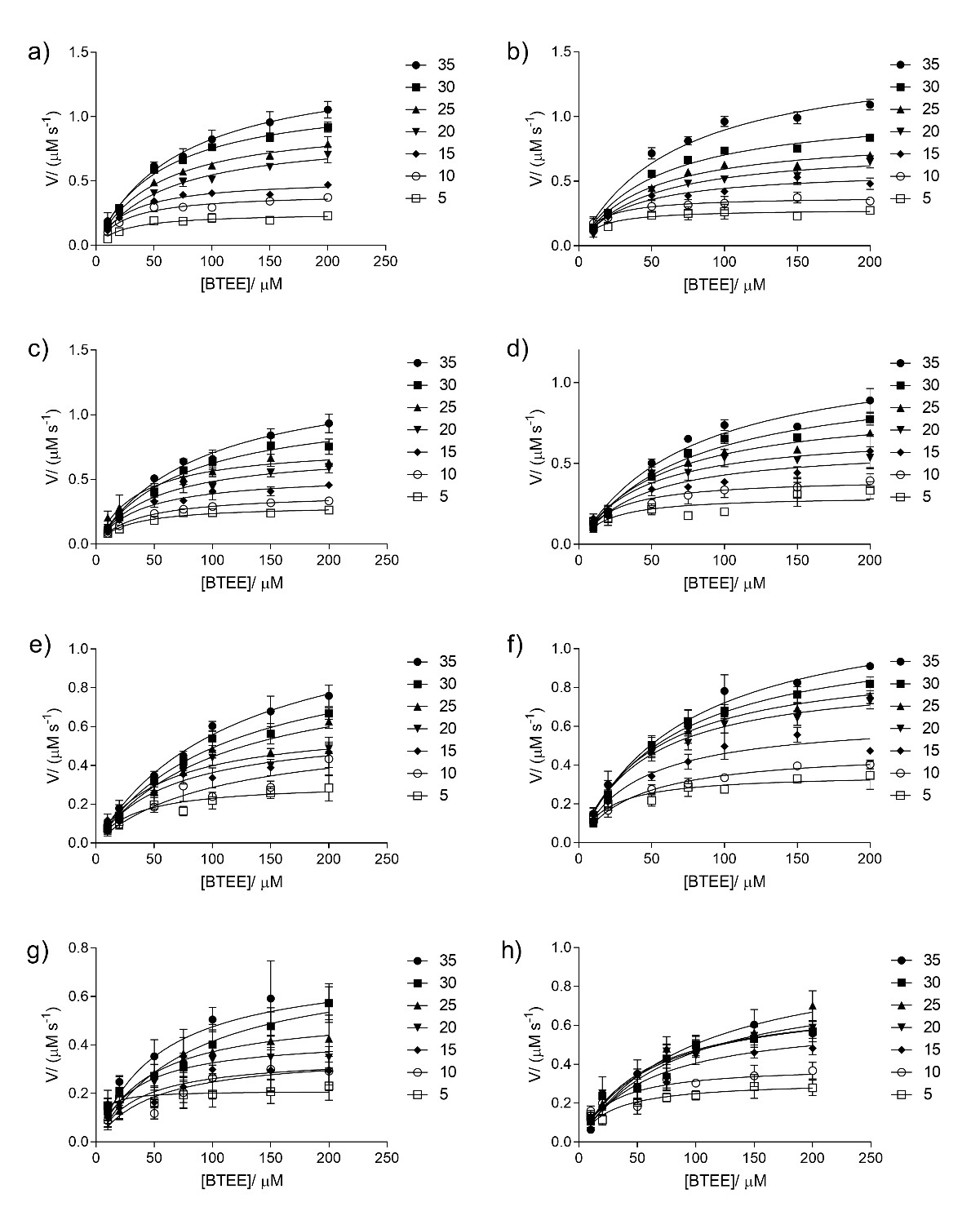


Figure S1: **Michaelis-Menten curves of α-chymotrypsin activity.** The activity of α-chymotrypsin from 35°C to 5°C in; a) buffer, b) buffer and 1 M glycine, c) 1 M NaClO_4_, d) 1 M NaClO_4_ and 1 M glycine, e) 0.25 M Mg(ClO_4_)_2_, f) 0.25 M Mg(ClO_4_)_2_ and 1 M glycine, g) 0.5 M Mg(ClO_4_)_2_, and h) 0.5 M Mg(ClO_4_)_2_ and 1 M glycine. Error bars represent the standard error of the mean with data points representing the mean of an *N*=4.


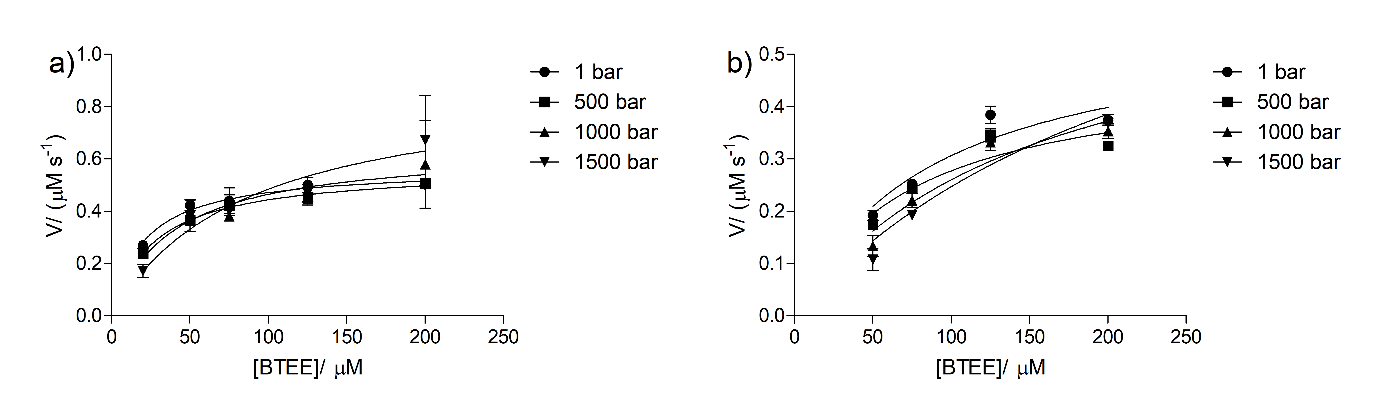


Figure S2: **Michaelis-Menten curves of α-chymotrypsin activity at high pressures**. The activity of α-chymotrypsin at 6 °C at high pressures in a) Tris buffer and b) 0.25 M Mg(ClO_4_)_2_. Error bars represent the standard error of the mean with data points representing the mean of an *N*=4.

|  | *k*_cat_ (s^-1^) | | | | | | |
| --- | --- | --- | --- | --- | --- | --- | --- |
|  | 308 K | 303 K | 298 K | 293 K | 288 K | 283 K | 278 K |
| Buffer | 71.23 | 58.88 | 48.77 | 43.50 | 25.89 | 20.33 | 12.66 |
| + glycine | 74.99 | 53.33 | 42.95 | 37.73 | 29.28 | 19.06 | 14.17 |
| 1 M NaClO_4_ | 67.47 | 54.43 | 38.36 | 36.13 | 26.80 | 19.70 | 15.02 |
| + glycine | 61.07 | 52.85 | 43.42 | 34.81 | 30.41 | 20.44 | 14.93 |
| 0.25 M Mg(ClO_4_)_2_ | 61.76 | 52.23 | 46.16 | 30.91 | 29.65 | 29.43 | 15.47 |
| + glycine | 62.55 | 54.75 | 47.66 | 43.60 | 32.13 | 24.05 | 17.73 |
| 0.5 M Mg(ClO_4_)_2_ | 36.58 | 37.67 | 26.90 | 21.51 | 17.61 | 18.32 | 10.54 |
| + glycine | 42.43 | 37.33 | 49.65 | 37.01 | 31.94 | 19.60 | 15.65 |

Table S1: **α-chymotrypsin k_cat_**. The turnover number (k_cat_) of α-chymotrypsin at all temperatures and conditions assayed. Kinetic parameters were determined from the Michaelis-Menten curves of N=4 replicates.

|  | *K*_M_ (μM) | | | | | | |
| --- | --- | --- | --- | --- | --- | --- | --- |
|  | 308 K | 303 K | 298 K | 293 K | 288 K | 283 K | 278 K |
| Buffer | 72.60 | 56.36 | 53.38 | 58.87 | 28.30 | 25.72 | 24.66 |
| + glycine | 67.41 | 51.72 | 44.56 | 44.08 | 31.79 | 13.14 | 12.80 |
| 1 M NaClO_4_ | 91.49 | 74.45 | 35.97 | 49.46 | 37.84 | 34.97 | 27.49 |
| + glycine | 77.48 | 73.72 | 55.99 | 41.82 | 41.97 | 21.94 | 18.43 |
| 0.25 M Mg(ClO_4_)_2_ | 121.3 | 113.1 | 108.0 | 55.19 | 63.49 | 105.40 | 34.37 |
| + glycine | 73.40 | 62.65 | 50.05 | 45.68 | 39.84 | 38.71 | 19.35 |
| 0.5 M Mg(ClO_4_)_2_ | 54.05 | 81.55 | 44.70 | 31.98 | 35.02 | 48.40 | 4.72 |
| + glycine | 83.04 | 59.91 | 97.13 | 56.40 | 57.65 | 25.03 | 25.67 |

Table S2: **α-chymotrypsin *K*_M_**. The Michaelis constant (*K*_M_) of α-chymotrypsin at all temperatures and conditions assayed. Kinetic parameters were determined from the Michaelis-Menten curves of *N*=4 replicates.

|  | *k*_cat_/*K*_M_ (M^-1^ s^-1^) | | | | | | |
| --- | --- | --- | --- | --- | --- | --- | --- |
|  | 308 K | 303 K | 298 K | 293 K | 288 K | 283 K | 278 K |
| Buffer | 9.81x10^5^ | 1.04x10^6^ | 9.14x10^5^ | 7.39x10^5^ | 9.15x10^5^ | 7.90x10^5^ | 5.13x10^5^ |
| + glycine | 1.11x10^6^ | 1.03x10^6^ | 9.64x10^5^ | 8.56x10^5^ | 9.21x10^5^ | 1.45x10^6^ | 1.11x10^6^ |
| 1 M NaClO_4_ | 7.37x10^5^ | 7.31x10^5^ | 1.07x10^6^ | 7.30x10^5^ | 7.08x10^5^ | 5.63x10^5^ | 5.46x10^5^ |
| + glycine | 7.88x10^5^ | 7.17x10^5^ | 7.75x10^5^ | 8.32x10^5^ | 7.25x10^5^ | 9.32x10^5^ | 8.10x10^5^ |
| 0.25 M Mg(ClO_4_)_2_ | 5.09x10^5^ | 4.62x10^5^ | 4.27x10^5^ | 5.60x10^5^ | 4.67x10^5^ | 2.79x10^5^ | 4.50x10^5^ |
| + glycine | 8.52x10^5^ | 8.74x10^5^ | 9.52x10^5^ | 9.54x10^5^ | 8.06x10^5^ | 6.21x10^5^ | 9.16x10^5^ |
| 0.5 M Mg(ClO_4_)_2_ | 6.77x10^5^ | 4.62x10^5^ | 6.02x10^5^ | 6.73x10^5^ | 5.03x10^5^ | 3.79x10^5^ | 2.23x10^6^ |
| + glycine | 5.11x10^5^ | 6.23x10^5^ | 5.11x10^5^ | 6.56x10^5^ | 5.54x10^5^ | 7.83x10^5^ | 6.10x10^5^ |

Table S3: **α-chymotrypsin *k*_cat_/*K*_M_.** The catalytic efficiency (*k*_cat_/*K*_M_) of α-chymotrypsin at all temperatures and conditions assayed. Kinetic parameters were determined from the Michaelis-Menten curves of *N*=4 replicates.

|  | Δ*G*^‡^ (kJ mol^-1^) | | | | | | |
| --- | --- | --- | --- | --- | --- | --- | --- |
|  | 308 K | 303 K | 298 K | 293 K | 288 K | 283 K | 278 K |
| Buffer | 64.63 | 64.02 | 63.39 | 62.56 | 62.70 | 62.14 | 62.07 |
| + glycine | 64.50 | 64.27 | 63.70 | 62.91 | 62.40 | 62.29 | 61.83 |
| 1 M NaClO_4_ | 64.77 | 64.22 | 63.98 | 63.02 | 62.61 | 62.21 | 61.70 |
| + glycine | 65.02 | 64.29 | 63.68 | 63.11 | 62.31 | 62.12 | 61.71 |
| 0.25 M Mg(ClO_4_)_2_ | 64.99 | 64.32 | 63.52 | 63.40 | 62.37 | 61.27 | 61.63 |
| + glycine | 64.96 | 64.20 | 63.45 | 62.56 | 62.18 | 61.74 | 61.32 |
| 0.5 M Mg(ClO_4_)_2_ | 66.34 | 65.14 | 64.86 | 64.28 | 63.62 | 62.38 | 62.52 |
| + glycine | 65.96 | 65.17 | 63.34 | 62.96 | 62.19 | 62.22 | 61.60 |

Table S4: **Free energy of activation of α-chymotrypsin**. The free energy of activation (ΔG^‡^) of α-chymotrypsin at all temperatures and conditions assayed.
